# Macrophage re-programming by JAK inhibitors relies on MAFB

**DOI:** 10.1007/s00018-024-05196-1

**Published:** 2024-03-25

**Authors:** Baltasar López-Navarro, Miriam Simón-Fuentes, Israel Ríos, María Teresa Schiaffino, Alicia Sanchez, Mónica Torres-Torresano, Alicia Nieto-Valle, Isabel Castrejón, Amaya Puig-Kröger

**Affiliations:** 1 Unidad de Inmunometabolismo e Inflamación, Instituto de Investigación Sanitaria Gregorio Marañón, Hospital General Universitario Gregorio Marañón, Madrid, Spain; 2 Myeloid Cell Laboratory, Centro de Investigaciones Biológicas, Madrid, Spain; 3 Unidad de Microscopía Confocal, Instituto de Investigación Sanitaria Gregorio Marañón, Hospital General Universitario Gregorio Marañón, Madrid, Spain; 4 Servicio de Reumatología, Instituto de Investigación Sanitaria Gregorio Marañón, Hospital General Universitario Gregorio Marañón, Madrid, Spain; 5 Departamento de Medicina, Universidad Complutense de Madrid, Madrid, Spain

**Keywords:** GM-CSF, JAK inhibitors, Macrophages, Macrophage reprogramming, MAFB, Monocytes, Rheumatoid arthritis, Upadacitinib

## Abstract

Monocyte-derived macrophages play a key pathogenic role in inflammatory diseases. In the case of rheumatoid arthritis (RA), the presence of specific synovial tissue-infiltrating macrophage subsets is associated with either active disease or inflammation resolution. JAK inhibitors (JAKi) are the first targeted synthetic disease-modifying antirheumatic drugs (tsDMARD) approved for treatment of RA with comparable efficacy to biologics. However, the effects of JAKi on macrophage specification and differentiation are currently unknown. We have analyzed the transcriptional and functional effects of JAKi on human peripheral blood monocyte subsets from RA patients and on the differentiation of monocyte-derived macrophages promoted by granulocyte-macrophage colony-stimulating factor (GM-CSF), a factor that drives the development and pathogenesis of RA. We now report that JAKi Upadacitinib restores the balance of peripheral blood monocyte subsets in RA patients and skewed macrophages towards the acquisition of an anti-inflammatory transcriptional and functional profile in a dose-dependent manner. Upadacitinib-treated macrophages showed a strong positive enrichment of the genes that define synovial macrophages associated to homeostasis/inflammation resolution. Specifically, Upadacitinib-treated macrophages exhibited significantly elevated expression of MAFB and MAFB-regulated genes, elevated inhibitory phosphorylation of GSK3β, and higher phagocytic activity and showed an anti-inflammatory cytokine profile upon activation by pathogenic stimuli. These outcomes were also shared by macrophages exposed to other JAKi (baricitinib, tofacitinib), but not in the presence of the TYK2 inhibitor deucravacitinib. As a whole, our results indicate that JAKi promote macrophage re-programming towards the acquisition of a more anti-inflammatory/pro-resolution profile, an effect that correlates with the ability of JAKi to enhance MAFB expression.

**Supplementary Information:**

The online version contains supplementary material available at 10.1007/s00018-024-05196-1.

## Introduction

Macrophages and macrophage-derived cytokines (TNFα, IL-6 and GM-CSF) have a major pathogenic role in rheumatoid arthritis (RA). Macrophages accumulate in the synovium of RA joints, where they exhibit destructive and remodeling effects and contribute to inflammation and joint destruction [1, 2]. Several multicenter and collaborative studies using single-cell omics technology have now illustrated the heterogeneity of active RA synovial tissue macrophages [3–5] in comparison with those from patients in remission and healthy subjects. Specifically, the monocyte-derived MerTK^neg^CD206^neg^ macrophage subset, that infiltrates the synovium and differentiates into macrophages along RA evolution, is specifically associated with RA pathology. Conversely, tissue-resident MerTK^pos^CD206^pos^TREM2^pos^ and FOLR2^high^/LYVE1^pos^ subsets, identified in the lining and sublining layers, respectively, are associated with homeostasis and remission [3, 6].

GM-CSF strongly influences the development and pathogenesis of RA [7]. Indeed, GM-CSF-deficient mice fail to develop joint pathology and associated pain in inflammatory arthritis models, and blockade of GM-CSF reduces the severity of established disease in wild type mice [8]. Besides driving tissue inflammation [7–9], GM-CSF is critical for the generation of lung alveolar macrophages, and primes monocytes for differentiating into macrophages (GM-MØ) with robust activin A-dependent antigen-presenting, immuno-stimulatory and pro-inflammatory [IL10^low^IL6^high^] activity. Unlike GM-CSF, M-CSF is indispensable for tissue-resident and monocyte-derived macrophage differentiation [10, 11], and primes monocytes to differentiate into macrophages (M-MØ) with a MAFB-dependent anti-inflammatory, reparative and immunosuppressive profile (IL10^high^IL6^low^) upon stimulation [11, 12]. Our group and others have shown that M-MØ and GM-MØ exhibit unique anti-inflammatory and pro-inflammatory transcriptional profiles [13–15], and that the transcriptome of GM-MØ strongly resembles that of pathogenic macrophages in RA [16, 17]. Given the role of GM-CSF in RA, a number of clinical trials targeting GM-CSF or GM-CSFR have been carried out and shown rapid and sustained clinical responses without major safety concerns [18, 19], and inhibitors of JAK2, a major downstream signaling effector of GM-CSFR, have also shown clinical efficacy in RA [20, 21].

JAKs are a family of protein tyrosine kinases associated with the cytoplasmic domain of type I and II cytokine receptors and become activated for intracellular signaling upon receptor engagement by their cognate ligands. The four JAK isoforms (JAK1, 2, 3 and TYK2) dimerize after receptor activation, become phosphorylated and activate specific signal transducers and activators of transcription (STATs), which subsequently induce gene transcription [20, 22]. The various JAK-STAT axes transmit the intracellular signaling initiated by numerous cytokines (IL-6, IL-23) as well as all types of interferons, hormones (EPO, GH) and growth factors (GM-CSF, G-CSF) and are critically involved in homeostatic (hematopoiesis) and pathological processes (antiviral response). Importantly, JAK-dependent cytokines like IL-6 and GM-CSF are major contributors to immunopathology in RA, and the blockade of their intracellular signaling is beneficial [20, 21]. Supporting the pathological role of JAK in RA, a recent GWAS study revealed a significant association between polymorphisms in the genes of the JAK-STAT signaling pathway (*TYK2*, *STAT4*, *IL6R*) and augmented risk for seropositive RA [23]. JAK inhibitors (JAKi) have shown efficacy in the management of RA both as monotherapy and in combination with Methotrexate (MTX), and are recommended following inadequate response to conventional or biologics treatments [24]. At present, five JAKi (Tofacitinib, Baricitinib, Filgotinib, Upadacitinib and Peficitinib) have received market approval or are undergoing clinical trials for RA treatment. Importantly, all JAKi have shown efficacy in the management of RA with comparable safety profile to other biological disease-modifying antirheumatic drugs (bDMARD) in both clinical trials and real-world [22, 25].

The effects of JAKi on macrophages have been mostly explored in terms of cytokine signaling and cytokine and chemokine production in response to danger signals [26, 27]. Tofacitinib and Baricitinib prevent GM-CSF-induced JAK2/STAT5 phosphorylation in THP-1 cells, and IL-1β production by neutrophils [27], and Baricitinib suppresses the in vivo production of cytokines by lung macrophages and limit the recruitment of neutrophils to the lung in SARS-CoV-2-infected macaques [28]. However, whether JAKi affects monocyte-to-macrophage differentiation or conditions macrophage re-programming has not been explored so far. In the present manuscript, we present evidence that JAK inhibitors re-program monocyte-to-macrophage differentiation in a dose-dependent manner and via enhanced expression of MAFB.

## Materials and methods

### Patients and flow cytometry

A total of three patients with rheumatoid arthritis (RA) and inadequate response to DMARD receiving Upadacitinib as indicated by the treating rheumatologist were invited to participated in this study. Nine normal donors with a mean (SD) age of 52 (11) years were also invited. Informed consent was obtained from these participants and the study was conducted in accordance with the Declaration of Helsinki, and approved by the Ethical Committee of Hospital General Universitario Gregorio Marañón (protocol code: JAKi-2022-v1 and ESCL_REUINM_2023). The three patients with seropositive RA were female with a mean (SD) age of 45 (15) years and moderate disease activity before initiating Upadacitinib treatment (rinvoq 15 mg daily) with a mean (SD) CRP based Disease Activity Score 28- joint counts (DAS28-CRP) of 4.1 (0.7). The three patients showed improvement at three months follow-up with a mean (SD) DAS28-CRP of 1.1 (0.2). A more detailed patient description is presented in Supplementary file. Blood was obtained in K_2_-EDTA tubes before and three months after Upadacitinib treatment. 100 µl of whole blood was labeled for 30 min. at 4 °C with the following antibodies obtained from Beckman Coulter CD45-Krome Orange (J33), CD14-PC7 (RM052), CD16-FITC (3G8), HLA-DR APCA750 (Immu-357). Following surface staining, cells were treated in BD Pharm Lyse (BD Biosciences) and IOTest 3 Fixative Solution (Beckman Coulter) according to the manufacturer’s instructions. Viability was determined with 7-aminoactinomycin D (7-AAD Viability Dye, Beckman Coulter) staining, and flow cytometry analysis performed using CytoFLEX S and Kaluza Analysis 2.1 software (Beckman Coulter). Monocyte subsets were gated on the basis of forward scatter (FSC) and side scatter (SSC) and CD14/CD16/HLA-DR expression as described in Supplementary Fig. 1.

### Cell culture

Human peripheral blood mononuclear cells (PBMC) were isolated from buffy coats from normal donors over a Lymphoprep (Nycomed Pharma) gradient. Monocytes were purified from PBMC by magnetic cell sorting using CD14 microbeads (Miltenyi Biotech). Monocytes were cultured at 0.5 × 10^6^ cells/ml for 7 days in RPMI 1640 supplemented with 10% fetal calf serum, at 37 °C in a humidified atmosphere with 5% CO_2_, and containing GM-CSF (1000 U/ml, ImmunoTools) to generate GM-CSF-polarized macrophages (GM-MØ) or M-CSF (20 ng/ml, ImmunoTools) to generate M-CSF-polarized macrophages (M-MØ). GM-CSF or M-CSF were added every two days. Based on the dosage and scheduled regime treatment of JAKi in RA patients (daily oral ingestion), Baricitinib and Upadacitinib (10–100 nM, Selleckchem) were added once-a-day during the 7-day differentiation procedure. The concentrations were selected based on enzyme assays (JAK2 IC50 = 5.7 nM for Baricitinib and JAK2 IC50 = 109 nM for Upadacitinib) and in vivo dosage [29]. In some experiments, JAKi were added to mature macrophages (on day 5 of the differentiation protocol, short-term treatment). Upadacitinib and Baricitinib were dissolved in fresh DMSO at 10 mM initial concentration, and control experiments were done by exposing macrophages to the same amount of DMSO (final concentration 0.001%). Viability of the resultant cell culture was assessed with 7-AAD staining. STAT5-IN-1 (50 µM, STAT5 phosphorylation specific inhibitor, Selleckchem) and U0126 (2,5 µM, MEK1/2 inhibitor, Selleckchem) were added to monocytes together with GM-CSF once-a-day during 2-days. LPS (10 ng/ml, 0111:B4 strain, Invivogen) was added at the indicated time points onto 7-day fully differentiated macrophages.

### RNAseq and GSEA

Total RNA was isolated from three independent preparations and processed at BGI (https://www.bgitechsolutions.com), where library preparation, fragmentation and sequencing were performed using the BGISEQ-500 platform. An average of 5.41 Gb bases were generated per sample and, after filtering, clean reads were mapped to the reference (UCSC Genome assembly hg38) using Bowtie2 (average mapping ratio 93.41%). Gene expression levels were calculated by using the RSEM software package. Differential gene expression was assessed by using DEseq2 algorithms using the parameters Fold change > 2 and adj*p* < 0.05. For gene set enrichment analysis (GSEA) [30], the gene sets available at the website, as well as gene sets generated from publicly available transcriptional studies, were used. The datasets used throughout the study are listed and described in Supplementary Table S1. The data reported in this publication have been deposited in NCBI's Gene Expression Omnibus and are accessible through GEO Series accession number GSE232044 (Upadacitinib-treated GM-MØ) and GSE253495 (Monocytes from Upadacitinib-treated RA patients). The prediction of transcription factor activities (DoRothEA) was done in R v4.2.0 with the packages Discriminant Regulon Expression Analysis [31], limma and BiocParallel. Principal Component Analysis (PCA) on the RNAseq from macrophages was preprocessed using the rlog function for normalization of read counts in R v4.2.0.

### Small interfering ribonucleic acid (siRNA) transfection

Monocytes (1 × 10^6^ cells) were transfected with human MAFB-specific siRNA (siGENOME siRNA SMARTpool, 25 nM) (Dharmacon) using Lipofectamine 3000 Transfection Reagent (ThermoFisher). Silencer™ Select Negative Control No. 1 siRNA (25 nM) (Dharmacon) was used as negative control siRNA. Six hours after transfection, cells were allowed to recover from transfection in complete medium, exposed to Upadacitinib (100 nM) for 42 additional hours, and lysed. Knock-down of MAFB was confirmed by Western blot.

### Quantitative real time RT-PCR

Total RNA was retrotranscribed and cDNA was quantified using the Universal Human Probe Roche library (Roche Diagnostics). Quantitative real-time PCR (qRT-PCR) was performed on a LightCycler^®^ 480 (Roche Diagnostics). Assays were made in triplicates and results normalized according to the expression levels of TBP. Results were obtained using the ΔΔCT method for quantitation. The oligonucleotides used to quantify mRNA transcripts were (5′–3′): LGMN forward: gaacaccaatgatctggagga; LGMN reverse: ggagacgatcttacgcactga; CD163 forward: gaagatgctggcgtgacat; CD163 reverse: gctgcctccacctctaagtc; CMKLR1 forward: cttgatgggaggcgtgac; CMKLR1 reverse: accgtaactgatggaagtgttg; FOLR2 forward: gagagaggccaactcagacac; FOLR2 reverse: ccagaccatgtctttctgtcc; MS4A6A forward: cggactgct atacagccaaag; MS4A6A reverse: tccagcagagtgcaaatcag; IL10 forward: tcactcatggctttgtagatgc; IL10 reverse: gtggagcaggtgaagaatgc; TBP forward: cggctgtttaacttcgcttc; TBP reverse: cacacgccaagaaacagtga.

### ELISA

Supernatants from GM-MØ were tested for the presence of IL-10, TNFα, IL-6 (Biolegend), activin A and LGMN (R&D Systems) and L-Lactate (Abcam) following the procedures supplied by the manufacturers.

### Phagocytosis

Phagocytosis ability was assessed by flow cytometry using pHrodo Red E. coli BioParticles Conjugates (Thermo Fisher), following the procedures recommended by the manufacturer. Macrophages were cultured in 24-well plate and exposed to pHrodo bioparticles for 60 min at 37 °C/5% CO_2_. Cells were then harvested and assessed by flow cytometry.

### Preparation of apoptotic cells and efferocytosis

Jurkat cells were cultured in RPMI 1640 medium without FCS for 16 h, and then treated with staurosporine (0.5 μg/ml, SIGMA), followed by an incubation at 37 °C for 3 h. Staurosporine treatment yielded a population of 83% annexin V + cells. Apoptotic cells (AC) were resuspended at a concentration of 1 × 10^6^ cells/ml and labeled with CellTrace™ Violet reagent (0.5 μM, Invitrogen, Thermo Scientific) for 20 min. For efferocytosis, macrophages were cultured with labeled AC (ratio 1:4) in p24 plates during 1 h at 37 °C. After 1 h, macrophages were rinsed with PBS to remove unbound AC and detached with PBS 5 mM EDTA, pelleted by centrifugation and fixed (IOTest 3 Fixative Solution, Beckman Coulter) before analyzing by flow cytometry.

### Video microscopy

Macrophages were seeded and cultured overnight in a collagen-coated surface (6 μg/ml, STEMCELL), and Jurkat cells (apoptotic or alive) previously labeled with CellTrace™ Violet (0.5 µM) were added to the culture at a proportion of 1:4 (macrophage:Jurkat) to image the efferocytosis process. Cells were live-imaged in a controlled stage at 37 °C and 5% CO_2_ with a sCMOS Orca Flash digital camera (Hamamatsu) coupled to a DMi8 microscope (Leica) for 2 h.

### Western-blot

Cell lysates were obtained in RIPA buffer containing 1 mM PIC (Protease Inhibitor Cocktail, SIGMA), 10 mM NaF, 1 mM Na_3_VO_4_ and 0.5 mM DTT. 10–30 µg cell lysate was subjected to SDS-PAGE and transferred onto an Immobilon polyvinylidene difluoride membrane (Millipore). For folate receptor beta (FOLR2), cell lysates were subjected to SDS-PAGE under non-reduced conditions. Protein detection was carried out using rabbit antibodies against pp38 and pERK (clones D3F9 and D13.14.4E, Cell Signaling, 1/1000), MAFB (HPA005653, Santa Cruz, 1/1000), pGSK3β (clone D85E12, Cell Signaling, 1/1000) and mouse monoclonal antibody against human CD163 (clone EDHu-1, Bio-Rad, 1/1000), pSTAT5 (clone 8-5-2, Millipore, 1/1000), FOLR2 (FRβ, kindly provided by Dr. Takami Matsuyama [32], dilution 1/800). Protein loading was normalized using an antibody against GAPDH (6C5, Santa Cruz Biotechnology, 1/2000) or against human vinculin (clone VIN-11-5, Sigma-Aldrich, 1/3000).

### Measurement of cellular respiration and extracellular acidification (bioenergetic profile)

The XF24 extracellular flux analyzer (Seahorse Biosciences, North Billerica, MA) was used to determine the bioenergetic profile of intact cells. Briefly, cells were seeded (200,000 cells/well) in XF24 plates (Seahorse Biosciences) and allowed to recover for 24 h. Cells were then incubated in bicarbonate-free DMEM (Sigma-Aldrich) supplemented with 11 mM glucose, 2 mM L-glutamine, 1 mM pyruvate, and 2% FBS in a CO_2_-free incubator for 1 h. The oxygen consumption rate (OCR) and extracellular acidification rate (ECAR), a proxy for lactate production, were recorded to assess the mitochondrial respiratory activity and glycolytic activity, respectively. After four measurements under basal conditions, cells were treated sequentially with 1 mM oligomycin, 0.6 mM carbonyl cyanide p-(trifluoromethoxy)phenylhydrazone (FCCP), 0.4 mM FCCP, and 0.5 mM rotenone plus 0.5 mM antimycin A (Sigma-Aldrich), with three consecutive determinations under each condition that were subsequently averaged. Basal respiration was estimated from the difference beetween the last rate measurement before first injection and the non-mitocondrial respiration rate. Non-mitochondrial respiration (OCR value after rotenone plus antimycin A addition) was subtracted from all OCR measurements. ATP turnover was estimated from the difference between the basal respiration and the oligomycin-inhibited respiration, and the maximal respiratory capacity was the rate in the presence of the uncoupler FCCP. In the case of ECAR parameters, the glycolysis capacity was obtained from the difference between the basal respiration and the oligomycin-inhibited respiration. The maximal glycolysis capacity was estimated from the average of the oligomycin-inhibited respiration. Six independent replicas of each analysis were done, and results were normalized to 1 µg of protein.

### Statistical analysis

Statistical analysis was done using GraphPad Prism, using parametric Student’s t test, as appropriate, and one-way ANOVA test coupled with Tukey´s post hoc test where indicated. Two-sided *p* value < 0.05 was considered significant (*p < 0.05; **p < 0.01, ***p < 0.001).

## Results

### Upadacitinib re-establishes the balance of peripheral blood monocyte subsets in RA

**Fig. 1:**
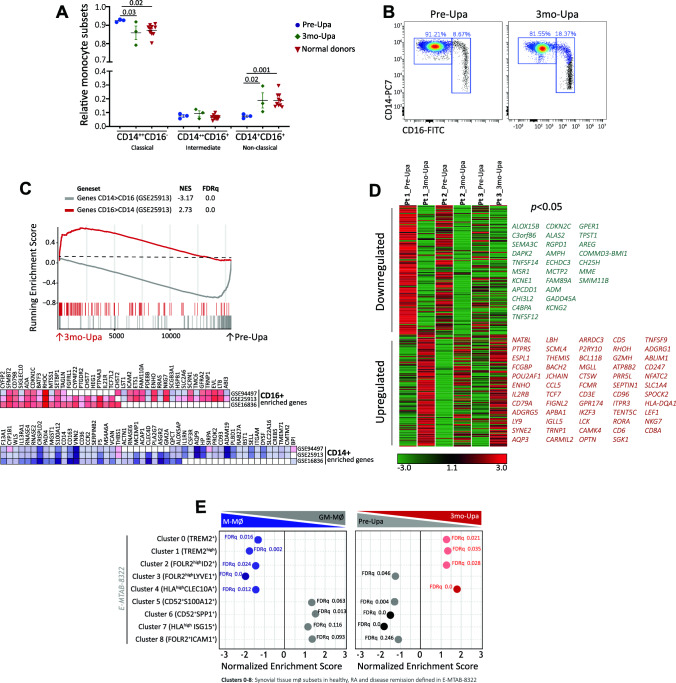
Upadacitinib re-establishes the balance of monocyte subsets in circulation **A** Relative frequency of monocyte subsets in three rheumatoid arthritis (RA) patients before (Pre-Upa) and three months after Upadacitinib treatment (3mo-Upa) and in nine normal donors sex-matched (*p* values are indicated, linear mixed models were used to examine monocyte subset changes between Pre-Upa vs 3mo-Upa; student's t-test for independent groups was used to detect monocyte subset changes in Pre-Upa and 3mo-Upa compared to normal donors). **B** A representative plot of CD14 and CD16 expression in one RA patient before and three months after Upadacitinib treatment. **C** GSEA on the ranked comparison of the 3 month Upadacitinib (3mo-Upa) treated versus pre-treated (Pre-Upa) monocyte transcriptomes, using the genes significantly overexpressed in CD16 + and in CD14 + monocytes (GSE25913) as data set. Normalized Enrichment Score (NES) and False Discovery Rate (FDRq) are indicated. Leading edge analysis of the GSEA of the genes that define the CD16 + or CD14 + monocyte subsets (GSE94497, GSE25913, GSE16836) on the ranked comparison of the transcriptomes of 3mo-Upa versus Pre-Upa monocytes is shown in the bottom panel. In the heatmap, expression values are represented as colors, where the range of colors (red, pink, light blue, dark blue) shows the range of expression values (high, moderate, low, lowest). **D** Heatmap of the expression of genes significantly (*p* < 0.05) altered by Upa treatment. For each gene, mRNA expression level is represented after normalizing gene expression and k-means clustering using Genesis (http://genome.tugraz.at/genesisclient/). The group of genes whose expression is either up-regulated or down-regulated by 25% after Upadacitinib treatment in the three patients is shown. **E** GSEA on the ranked comparison of the GM-MØ versus M-MØ transcriptomes (left) and the ranked comparison of the 3 month upadacitinib (3mo-Upa) treated versus pre-treated (Pre-Upa) monocyte transcriptomes (right), using the genes preferentially expressed by RA-specific clusters of synovial tissue macrophage (E-MTAB-8322) as data set. NES and FDRq value are indicated (FDRq < 0.01, dark filled circle; FDRq > 0.250, empty circle). The intensity of color increases with the enrichment of the gene signature

To assess whether JAK inhibitors (JAKi) had an effect on myeloid cells in RA patients, we initially determine the relative levels of monocyte subsets in peripheral blood before and after a 3-month period of Upadacitinib (Upa) treatment. Analysis of 3 independent RA patients showed that Upa treatment results in significantly diminished levels of the classical CD14 + + CD16- monocyte subset and an augmented proportion of non-classical CD14 + CD16 + monocytes (Fig. 1A–B and Supplementary Fig. 1). Moreover, after three months of treatment, monocyte subsets levels of RA patients were similar to those found in normal donors (Fig. 1A). Since reduction of the non-classical CD14 + CD16 + monocyte subset characterizes peripheral blood from active RA patients [33], these results indicate that Upa restores the balance of monocyte subsets found in healthy individuals, a finding that agrees with the known therapeutic action of JAKi (Fig. 1A–B). Transcriptomic analysis of CD14 + monocytes isolated from RA patients before (Pre-Upa) and 3 months along Upa treatment (3mo-Upa) further confirmed these phenotypic changes. GSEA revealed that the transcriptome of 3mo-Upa has a very significant over-representation of the genes that define non-classical (CD16 +) monocytes as well as a significantly reduced expression of the genes that define classical (CD14 +) monocytes, using the genesets previously defined GSE25913 [34], GSE94497 [35], and GSE16836 [36] (Fig. 1C). In fact, leading edge analysis evidenced that, besides CD16 (*FCGR3A*), Upadacitinib upregulates numerous other genes that characterize CD16 + monocytes, including *RHOC* and *ADA*. Conversely, Upadacitinib downregulates not only paradigmatic CD14 + monocyte marker genes (*CD14, CCR2*) but also genes whose expression characterizes CD14 + classical monocytes (*VNN2, S100A12, SERPINB2*) (Fig. 1C). Specifically, comparison of the transcriptome of Pre-Upa and 3mo-Upa indicated the existence of 149 genes whose expression is significantly (*p* < 0.05) diminished by Upa treatment, including STAT-dependent genes like *PIM1*, *IFIT1* and *RSAD2* [35, 37, 38] (Fig. 1D, Supplementary Table S1). On the other hand, the 129 genes whose expression is augmented upon Upa treatment included several genes associated to anti-inflammatory functions like *CD28* and *CD127* [39, 40] (Fig. 1D, Supplementary Table S1). Altogether, these findings indicate that JAKi influence monocyte differentiation at the phenotypic and transcriptional level.

JAKi ameliorate the signs and symptoms of RA and are currently used for the treatment of RA [20, 21]. Recent research has shed light on the transcriptome of subsets of synovial tissue macrophages (STMs) across various states: in healthy subjects, RA patients, and those in RA remission [3]. In light of this, we investigated the impact of Upa on the expression of gene clusters defining distinct STM subsets. Notably, the gene clusters that define the four pathogenic macrophage subsets in RA, and whose transcriptome resembles that of GM-MØ (Clusters 5–8, Fig. 1E), were extremely responsive to Upa treatment, as their expression was significantly diminished in 3mo-Upa (Fig. 1E). Conversely, the expression of the gene clusters that define four of the five STM subsets associated to homeostasis and/or remission (Clusters 0–4, Fig. 1E), which exhibit a transcriptome akin to anti-inflammatory M-MØ, were increased in 3mo-Upa (Fig. 1E). As a whole, treatment with Upa causes CD14 + monocytes from RA patients to exhibit reduced expression of genes associated to pathogenic STM subsets, and, concurrently, to acquire the expression of genes characterizing macrophages from RA patients in remission. These observations align well with the established therapeutic action of JAKi.

### Upadacitinib promotes the generation of monocyte-derived macrophages with an anti-inflammatory transcriptional and functional profile

**Fig. 2:**
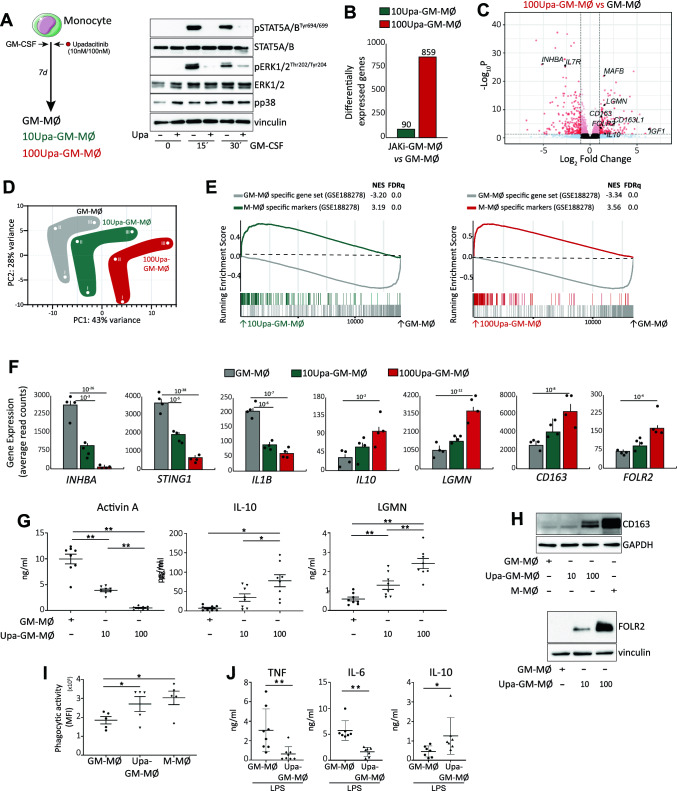
Upadacitinib promotes monocyte-derived macrophages with an anti-inflammatory gene expression and functional profile **A** Schematic representation of the experiments. Monocytes were exposed to 10–100 nM Upadacitinib daily during macrophage differentiation process with GM-CSF and the RNA levels were determined at day 7 on GM-MØ, 10Upa-GM-MØ and 100Upa-GM-MØ. Right, immunoblot analysis of pSTAT5, STAT5, pERK, ERK and pp38 by monocytes treated for 1 h to 100 nM Upadacitinib (Upa) and exposed to GM-CSF for the indicated time points. **B** Number of annotated genes whose expression is regulated in GM-MØ after 7d of Upadacitinib treatment (adj*p* < 0.05). **C** Volcano plot of RNAseq results showing the 100 nM Upadacitinib-induced gene expression changes in GM-MØ. **D** PCA analysis of GM-MØ, 10Upa-GM-MØ and 100Upa-GM-MØ.Three independent donors are identified as I, II and III. **E** GSEA on the ranked comparison of the GM-MØ versus 10Upa-GM-MØ and GM-MØ versus 100Upa-GM-MØ transcriptomes, using the genes significantly modulated by GM-CSF (GM-MØ-specific markers) and M-CSF (M-MØ-specific markers) as data set. Normalized Enrichment Score (NES) and False Discovery Rate (FDRq) are indicated. **F** Relative expression of the indicated genes as determined by RNA-sequencing on GM-MØ, 10Upa-GM-MØ and 100Upa-GM-MØ. Mean ± SEM of 4 independent donors are shown, with the indication of the *P*_adj_. **G** Production of activin A, IL-10 and LGMN by GM-MØ, 10Upa-GM-MØ and 100Upa-GM-MØ. Mean ± SEM of 8 independent donors are shown (*p < 0.05, **p < 0.01, one-way ANOVA with Tukey´s post hoc test; F = 92.63 for Activin A, F = 17.85 for IL-10, F = 33.39 for LGMN). **H** Immunoblot analysis of CD163 and FOLR2 (down) by GM-MØ, 10Upa-GM-MØ, 100Upa-GM-MØ and monocytes differentiated with M-CSF (M-MØ). In panels A-G, vinculin or GAPDH protein levels were determined as protein loading controls and a representative experiment of two independent donors is shown. **I** Phagocytic activity in GM-MØ, 100Upa-GM-MØ and M-MØ**.** Mean ± SEM of 5 independent donors are shown (*p < 0.05, one-way ANOVA with Tukey’s post hoc test, F = 13.74). **J** Production of TNFα, IL-6 and IL-10 by GM-MØ and 100Upa-GM-MØ challenged with LPS for 24 h, as determined by ELISA. Mean ± SEM of 7–8 independent donors are shown (*p < 0.05, **p < 0.01, paired t-test)

The phenotypic and transcriptional effects of Upa treatment on human peripheral blood monocytes led us to hypothesize that JAKi might also modify the differentiation of monocyte-derived macrophages, which drive pathogenesis in most inflammatory diseases [41]. To directly address this hypothesis, we evaluated the effect of JAKi on the differentiation of monocyte-derived macrophages in response to GM-CSF, a major pathogenic cytokine in RA [7, 10]. To that end, 10 nM or 100 nM Upadacitinib (Upa) was added each day along the GM-CSF-dependent monocyte-to macrophage (GM-MØ) differentiation process to generate 10Upa-GM-MØ or 100Upa-GM-MØ (Fig. 2A). These Upa concentrations, which fall within the range of Upa levels found in Upa-treated RA patients [29], had no effect on macrophage viability (Supplementary Fig. 2A), and drastically impaired the GM-CSF-induced JAK2-dependent STAT5 and ERK phosphorylation in monocytes [42] (Fig. 2A). RNAseq revealed that 100 nM Upa not only reduces the expression of STAT5-dependent genes like *CISH* and *PIM1* [37, 38] (Supplementary Fig. 2B) but promotes a huge shift in the GM-MØ transcriptional profile. Specifically, 100Upa-GM-MØ exhibited significantly (|log_2_FC|> 1; *adjp* < 0.05) altered expression of 859 genes (347 genes upregulated, 512 genes downregulated) compared to control GM-MØ (Fig. 2B, C), whereas 10Upa-GM-MØ only showed 90 differentially expressed genes (Fig. 2B). Therefore, long-term Upa treatment modifies the acquisition of the transcriptional profile of GM-CSF-dependent monocyte-derived macrophages in a dose-dependent manner (Fig. 2B, D), an effect that concurs with its dose-dependent ability to inhibit the GM-CSF-induced intracellular signaling (Supplementary Fig. 2C). Importantly, gene ontology analysis of the transcriptome of 10Upa-GM-MØ and 100Upa-GM-MØ revealed that the presence of Upa significantly (FDRq = 0.0) diminishes the expression of the pro-inflammatory “GM-MØ-specific” gene set (GSE188278) [43] (Fig. 2E), which includes genes like *INHBA* (Fig. 2F), and reduces the production of *INHBA*-encoded activin A (Fig. 2G), whose expression is particularly high in synovial macrophages from RA patients [16, 17]. Therefore, Upadacitinib weakens the expression of genes that characterize GM-CSF-dependent pro-inflammatory macrophages.

**Fig. 3:**
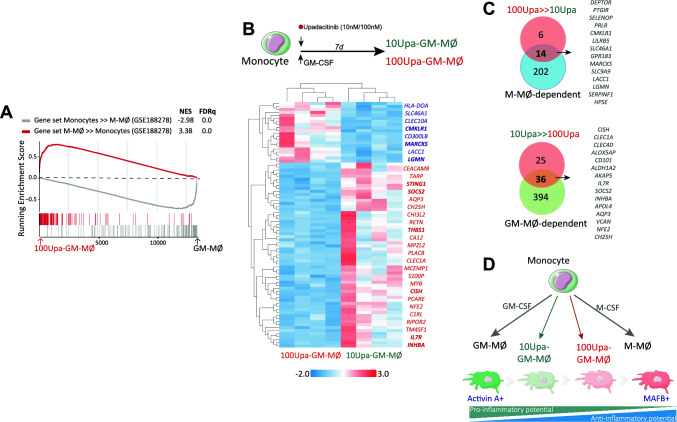
Dose-dependent effect of Upadacitinib on monocyte differentiation **A** GSEA on the ranked comparison of the GM-MØ versus 100Upa-GM-MØ transcriptome, using the genes significantly modulated along the monocyte-to-M-MØ differentiation as data set. NES and FDRq value are indicated. **B** Heatmap of the expression of genes significantly (|log2FC|> 1; *p* < 0.05) altered by Upadacitinib in 10Upa-GM-MØ and 100Upa-GM-MØ, as determined by RNAseq, data is represented as read counts standardized into z-score. **C** Comparison of genes differentially expressed in the indicated macrophage types. **D** Schematic representation of the dose-dependent effect of Upadacitinib on monocyte-to macrophage differentiation

Noteworthy, GSEA revealed an additional and unexpected effect of Upadacitinib, as the transcriptome of 10Upa-GM-MØ and 100Upa-GM-MØ exhibited a very significant over-representation of the genes that define anti-inflammatory monocyte-derived macrophages, namely, the M-CSF-dependent “M-MØ-specific” gene set (GSE188278) [43] (Fig. 2E), including *IL10, LGMN, CD163* and *FOLR2* (Fig. 2F, Supplementary Fig. 2D). Moreover, this anti-inflammatory effect of Upadacitinib was evident at the protein level, since Upa-treated macrophages had increased expression of IL-10, Legumain (LGMN), CD163 and FOLR2 (Fig. 2G, H). When compared to control GM-MØ, 100Upa-GM-MØ exhibited higher phagocytic activity and, higher levels of IL-10 and lower production of TNFα and IL-6 after exposure to LPS (Fig. 2I, J) (Supplementary Fig. 2E). All these results demonstrate that Upadacitinib not only limits the pro-inflammatory nature of GM-CSF-dependent monocyte-derived macrophages, but prompts the acquisition of anti-inflammatory features. Since the effect of Upa resembles the pro-differentiation action of M-CSF on monocytes (Fig. 2E–H), we also checked whether Upa modifies the expression of genes specifically regulated during monocyte-to-M-MØ differentiation (GSE188278). As shown in Fig. 3A, the genes exclusively upregulated along monocyte-to-M-MØ differentiation (“M-MØ >  > Monocytes”) were significantly over-expressed in 100Upa-GM-MØ, whose transcriptome also showed a reduced expression of the genes exclusively downregulated along monocyte-to-M-MØ (“Monocytes >  > M-MØ”, Fig. 3A). These results confirm the link between Upadacitinib and M-CSF-driven responses, and suggests that Upadacitinib re-programs macrophages at the transcriptional and functional level.

### The effect of upadacitinib on monocyte differentiation is dose-dependent

As 10Upa-GM-MØ and 100Upa-GM-MØ showed different protein levels of FOLR2, CD163, Legumain and IL-10 (Fig. 2G–H), we assessed whether the transcriptional effect of Upa on macrophages was dose dependent. Comparison of the gene profile of 10Upa-GM-MØ and 100Upa-GM-MØ identified 81 differentially expressed genes (Fig. 3B). Of note, 70% of the genes with higher expression in 100Upa-GM-MØ (14 out of 20) belong to the “M-MØ-specific” gene set, while 59% of the genes with higher expression in 10Upa-GM-MØ (36 out of 61) belong to the pro-inflammatory “GM-MØ-specific” gene set (Fig. 3C). Therefore, long-term Upadacitinib treatment dose-dependently allows for the acquisition of an M-CSF-dependent profile (Fig. 3D).

### Upadacitinib modulates the expression of genes that define macrophage subsets relevant in RA and tissue-resident macrophages

**Fig. 4:**
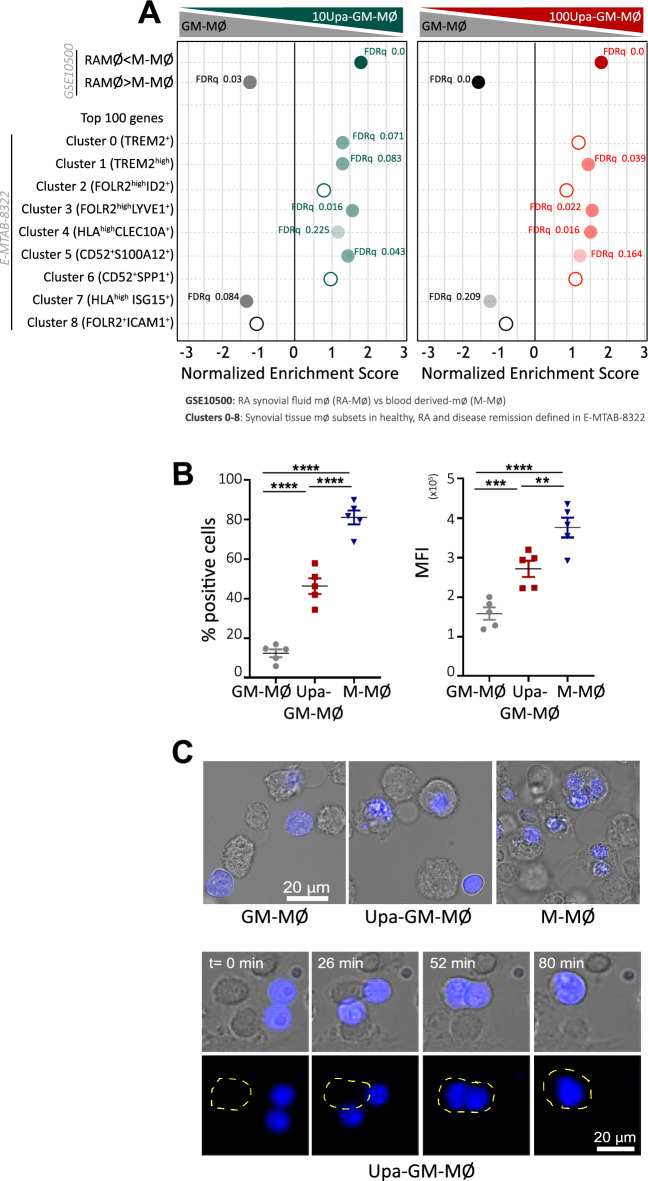
Upadacitinib modulates the expression of genes that define macrophage subsets relevant in RA **A** GSEA on the ranked comparison of the GM-MØ versus 10Upa-GM-MØ and GM-MØ versus 100Upa-GM-MØ transcriptomes, using the genes preferentially expressed by control or RA-specific macrophages (GSE10500) and RA-specific clusters of synovial tissue macrophage (E-MTAB-8322) as data set. NES and FDRq value are indicated (FDRq < 0.01, dark filled circle; FDRq > 0.250, empty circle). The intensity of color increases with the enrichment of the gene signature. **B** Efferocytosis (% positive cells and mean fluorescence intensity) of GM-MØ, 100Upa-GM-MØ and M-MØ as determined by flow cytometry using staurosporine-induced CellTrace Violet-labeled apoptotic Jurkat cells. Mean ± SEM of 5 independent donors are shown (**p < 0.01; ***p < 0.001, ****p < 0.0001, one-way ANOVA with Tukey´s post hoc test, F = 137.2 for % positive cells, F = 70.32 for MFI). **C** Above, representative images of macrophages (GM-MØ, 100Upa-GM-MØ or M-MØ) and apoptotic Jurkat cells (blue) after 1 h of co-culture, as indicated. Below, time-lapse of 100Upa-GM-MØ co-cultured with apoptotic Jurkat cells (blue) imaged for 120 min at 2-min intervals. Bright field images correspond to indicated time-lapse frames of co-culture. The macrophage shape is encircled in yellow to show its position and dynamics during the process. Bars, 20 µm

Since previous results have identified a set of genes preferentially expressed by macrophages from the synovium of RA patients (RAMØ) (GSE10500) [44], we next checked whether Upa affects the expression of genes preferentially expressed by RAMØ. GSEA revealed that RAMØ-specific genes are under-represented in the transcriptome of both 10Upa-GM-MØ and 100Upa-GM-MØ (Fig. 4A), thus indicating the inhibitory effect of Upadacitinib on the expression of genes that characterize pathogenic macrophages in RA. We also took advantage of recently published information on synovial tissue macrophage (STM) from RA patients [3], and analyzed the expression of gene clusters that are specific for the distinct STM subsets in 100Upa-GM-MØ. The gene profile of 100Upa-GM-MØ showed an over-representation of the genes that define the homeostatic STM MERTK^pos^ TREM2^high^ and FOLR2^high^/LYVE1^pos^ subsets, and also the tissue-infiltrating antigen presenting HLA^high^/CLEC10A^pos^ subset (Fig. 4A) [3, 6]. Reinforcing their homeostatic profile, TREM2^high^ and FOLR2^high^/LYVE1^pos^ subsets very significantly overexpress the gene sets that define anti-inflammatory/reparative M-MØ (Fig. 1E). Of note, and although to a lower extent, this enrichment was also seen in 10Upa-GM-MØ (Fig. 4A), further emphasizing the dose-dependent action of Upa. Consequently, exposure to Upadacitinib impairs the acquisition of the transcriptional profile of pathogenic RAMØ and also prompts the expression of genes that define STM clusters primarily involved in homeostasis and resolution of inflammation. At the functional level, 100Upa-GM-MØ exhibited higher efferocytosis than GM-MØ (Fig. 4B–C, Supplementary Fig. 2F and video microscopy) an anti-inflammatory activity related to MERTK^pos^ STM subsets [3] and the protective role of the MerTK pathway in joint pathology [45]. In agreement with these findings, analysis of the MoMac-VERSE (a resource that identifies conserved monocyte and macrophage states and global imprinting across human tissues) [46] (GSE178209) revealed that the transcriptome of 100Upa-GM-MØ is significantly enriched in the gene cluster that defines the macrophage HES1_Mac cluster 2, that exhibits a “long-term resident”-like macrophage signature (GSE188647), as well as genes that characterize tissue-resident macrophages from various tissues and organs, including *LGMN*, *MS4A6A* and *MAFB* [47] (Supplementary Fig. 3A–C).

### The macrophage re-programming effect of Upadacitinib relies on the MAFB transcription factor

**Fig. 5:**
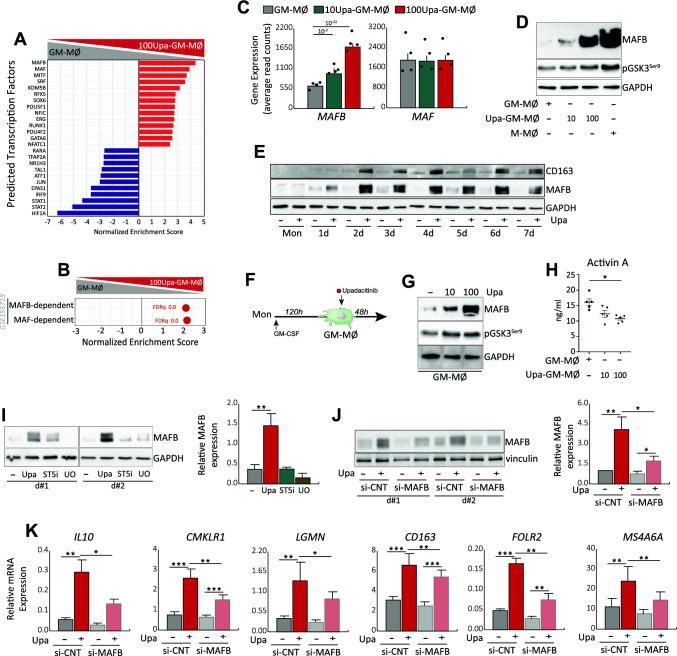
JAKi increases the expression of MAFB transcription factor in macrophages **A** Discriminant regulon expression analysis (DoRothEA) of 100Upa-GM-MØ compared with GM-MØ. Top 25 transcription factors are shown. **B** GSEA of genes downregulated by siRNA MAFB and by siRNA MAF on macrophages (GSE155719) on the ranked comparison of the transcriptomes of 100Upa-GM-MØ and GM-MØ transcriptomes. NES and FDRq value are indicated. **C** Relative expression of *MAFB* and *MAF* as determined by RNA-sequencing on GM-MØ, 10Upa-GM-MØ and 100Upa-GM-MØ. Mean ± SEM of 4 independent donors are shown, with the indication of the *P*_adj_. **D** Immunoblot analysis of MAFB and pGSK3S9 by GM-MØ, 10Upa-GM-MØ, 100Upa-GM-MØ and M-MØ. **E** Immunoblot analysis of MAFB and CD163 along the monocyte to macrophage differentiation in the presence of 100 nM Upadacitinib (Upa). In panels (D-E), GAPDH protein levels were determined as protein loading control and a representative experiment of two (**E**) and four (**D**) independent donors is shown. **F** Schematic representation of the experiments: short term-Upadacitinib treatment to mature macropahges (GM-MØ). Immunoblot analysis of MAFB and pGSK3S9 (**G**) and production of activin A (**H**) by GM-MØ exposed to 10–100 nM Upadacitinib for the last 48 h. GAPDH protein levels were determined as protein loading control. In (**G**) a representative experiment of three independent donors is shown. In (**H**) mean ± SEM of 5 independent donors are shown (*p < 0.05, F = 12.28). **I** Immunoblot analysis of MAFB in two independent preparations of differentiating GM-MØ (day 2) generated from monocytes exposed to DMSO (−), Upadacitinib (Upa, 100 nM), STAT5 phosphorylation specific inhibitor (ST5i, 50 µM) or MEK1/2 inhibitor (UO, 2,5 µM). Right, quantification of MAFB expression. **J** Immunoblot analysis of MAFB in two independent preparations of differentiating GM-MØ (day 2) generated from monocytes transfected with either siCNT or MAFB-specific siRNA (siMAFB) and exposed to DMSO (−) or 100 nM Upadacitinib (+). Right, quantification of MAFB expression. In panels I-J, mean ± SEM of the relative MAFB protein levels in the macrophage subtypes from four independent donors are shown (*p < 0.05, **p < 0.01). **K** Relative mRNA expression of the indicated MAFB-dependent genes in siCNT GM-MØ, siMAFB GM-MØ, siCNT Upa-GM-MØ and siMAFB Upa-GM-MØ (day 2). Mean ± SEM of four independent experiments are shown (*p < 0.05; **p < 0.01; ***p < 0.001, F = 3.7 for *IL10*, F = 6.8 for *CMKLR1*, F = 4.4 for *LGMN*, F = 56.89 for *CD163*, F = 9.24 for *FOLR2*, F = 8.34 for *MS4A6A*)

To identify the molecular basis of the macrophage re-programming effect of Upadacitinib we initially used Discriminant Regulon Expression Analysis (DoRothEA) [31]. The transcriptome of 100Upa-GM-MØ showed a negative enrichment in STAT1 and STAT2 regulons (Fig. 5A), as expected from the inhibitory effects of JAKi, and also exhibited diminished expression of the HIF1A-regulon, a result corroborated by the lower lactate release from 100Upa-GM-MØ cells, which also exhibited diminished levels of HIF1-regulated genes like *SLC2A1*, *EGLN3* and *AQP3* (Supplementary Fig. 4A–B) [48]. Importantly, Upadacitinib modified metabolic parameters in GM-MØ, including both glycolytic capacity and maximal ATP production (Supplementary Fig. 4C–G), indicating that JAKi alters not only the inflammatory but also the metabolic state in macrophages.

Conversely, DoRothEA revealed that the gene profile of 100Upa-GM-MØ is highly enriched in MAFB and MAF regulons (Fig. 5A), in agreement with the gene ontology data (Supplementary Fig. 3) and overexpresses the “M-MØ-specific” gene set [43] (Fig. 2E), whose M-CSF-driven acquisition is MAFB/MAF-dependent [10, 12, 49, 50]. Supporting the significance of these findings, the transcriptome of 100Upa-GM-MØ showed a high over-representation of MAFB- and MAF-dependent genes (Fig. 5B). As *MAFB* gene expression was also enhanced in 100Upa-GM-MØ (Fig. 5C), we next assessed MAFB protein level in macrophages generated in the presence of Upa. As shown in Fig. 5D, 100Upa-GM-MØ exhibited elevated MAFB protein levels as well as enhanced levels of inactive (Ser9-phosphorylated) GSK3β, whose active form limits MAFB protein and activity [51]. The higher expression of MAFB was observed at all time points along 100Upa-GM-MØ differentiation (Fig. 5E) and matched with a progressive enhancement of the expression of the MAFB-dependent protein CD163 (Fig. 5E). Therefore, the macrophage re-programming activity of Upadacitinib coincides with a dose-dependent increase in the expression of MAFB and MAFB-targets [12]. In fact, short-term exposure to Upa (two doses in the last 48 h of differentiation of GM-MØ) (Fig. 5F) sufficed to increase the expression of MAFB, enhance the inhibitory phosphorylation of GSK3β (Fig. 5G) and reduce activin A production (Fig. 5H), further reinforcing the link between the re-programming action of Upadacitinib and MAFB.

As blocking JAK2 activation with Upadacitinib results in impaired GM-CSF-induced STAT5 and ERK phosphorylation (Fig. 2A), we next compared MAFB expression in GM-CSF-primed monocytes exposed to Upa or known inhibitors of STAT5 and ERK. Interestingly, Upa treatment results in a stronger MAFB expression than STAT5 or ERK-activating inhibitors (Fig. 5I), suggesting that, besides STAT5 and ERK, JAK2 might trigger additional intracellular signaling directly involved in controlling the anti-inflammatory differentiation of human macrophages. In this regard, and to determine whether MAFB mediates the macrophage re-programming action of Upadacitinib, we evaluated the effect of Upa after siRNA-mediated MAFB knock-down in monocytes. Importantly, knock-down of MAFB (Fig. 5J) significantly reduced the positive effect of Upa on the expression of *IL10, CMKLR, LGMN, CD163, FOLR2* and *MS4A6A* (Fig. 5K). Therefore, MAFB mediates the reprogramming action of Upadacitinib during the GM-CSF-dependent differentiation of human monocyte-derived macrophages.

### Macrophage re-programming by other JAK inhibitors

**Fig. 6:**
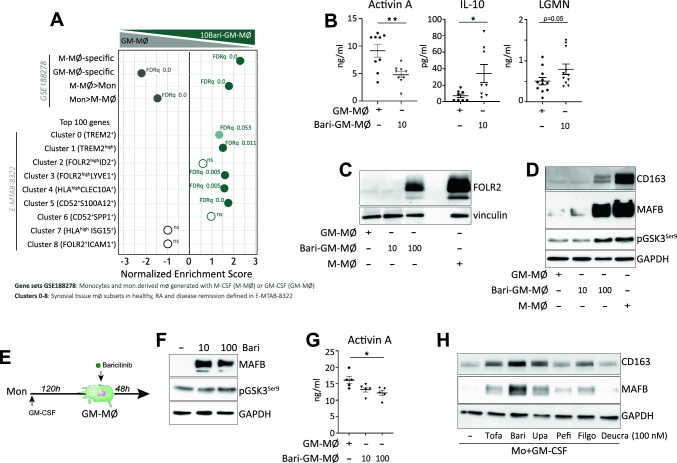
Macrophage re-programming by other JAK inhibitors **A** Monocytes were exposed to 10 nM Baricitinib daily during macrophage differentiation process with GM-CSF and the RNA levels were determined at day 7 on GM-MØ and 10Bari-GM-MØ. GSEA on the ranked comparison of the GM-MØ versus 10Bari-GM-MØ transcriptomes, using the genes preferentially expressed by GM-CSF (GM-MØ-specific) and M-CSF (M-MØ-specific) (GSE188278) and RA-specific clusters of synovial tissue macrophages (E-MTAB-8322) as data set. NES and FDRq value are indicated (FDRq < 0.01, dark filled circle; FDRq > 0.250, empty circle). **B** Production of activin A, IL-10 and LGMN by GM-MØ and 10Bari-GM-MØ. Mean ± SEM of 8–9 independent donors are shown (*p < 0.05, **p < 0.01, one-way ANOVA with Tukey´s post hoc test). **C**–**D** Immunoblot analysis of FOLR2 (**C**) MAFB, CD163 and pGSK3S9 (**D**) by GM-MØ, 10Bari-GM-MØ, 100Bari-GM-MØ and monocytes differentiated with M-CSF (M-MØ). Vinculin and GAPDH protein levels were determined as protein loading control. A representative experiment of two (**C**) and four (**D**) independent donors is shown. **E** Schematic representation of the experiments: short-term Baricitinib treatment to GM-MØ. Immunoblot analysis of MAFB and pGSK3S9 (**F**) and production of activin A (**G**) by GM-MØ exposed to 10–100 nM Baricitinib for the last 48 h. GAPDH protein levels were determined as protein loading control. In (**F**) a representative experiment of three independent donors is shown. In (**G**) mean ± SEM of 5 independent donors are shown (*p < 0.05, F = 7.26). **H** Immunoblot analysis of MAFB and CD163 in GM-MØ (day 2) generated from monocytes exposed to DMSO (−) or 100 nM Tofacitinib (Tofa), Baricitinib (Bari), Upadacitinib (Upa), Peficitinib (Pefi), Filgotinib (Filgo) or Deucravacitinib (Deucra). A representative experiment of two independent donors is shown

To date, five different JAKi (Tofacitinib, Baricitinib, Upadacitinib, Peficitinib and Filgotinib) have been approved for the treatment of RA [20, 24]. Given the re-programming action of Upadacitinib, we next asked whether other JAKi also exhibit a similar effect on the GM-CSF-dependent monocyte-derived macrophages. After checking for minimal effects on cell viability (Supplementary Fig. 2A), monocytes were exposed to different concentrations of Baricitinib (Bari, JAK1/2 inhibitor), Tofacitinib (Tofa, JAK1-3 inhibitor), Peficitinib (JAK3 inhibitor), Filgotinib (JAK1 inhibitor) and the TYK2 inhibitor Deucravacitinib each day along the monocyte-to macrophage differentiation process. Like Upa, long-term Bari treatment enhanced expression of the anti-inflammatory “M-MØ-specific” gene set, diminished expression of the pro-inflammatory “GM-MØ-specific” gene set, and augmented the expression of genes that define the tissue-resident homeostatic TREM2^high^ and FOLR2^high^LYVE1^pos^ subsets, as well as tissue-infiltrating antigen presenting HLA^high^/CLEC10A^pos^ subsets (Fig. 6A), from RA patients [3]. Altogether, these results confirmed that JAKi (Upa and Bari) favor the acquisition of homeostatic/resolution properties exhibited by macrophages in vivo. Besides, Bari-treated macrophages showed diminished expression of activin A and increased expression of IL-10, LGMN, CD163 and FOLR2 (Fig. 6B–D). More importantly, Bari dose-dependently increased MAFB and GSK3β Ser9 phosphorylation, and reduced activin A production, both after long-term (Fig. 6D) or short-term (Fig. 6E–G) treatment in mature macrophages, hence indicating its net anti-inflammatory effect. Of note, clinically-used doses of Tofacitinib, Peficitinib and Filgotinib also increased the expression of CD163 and MAFB, an effect not seen with Deucravacitinib (Fig. 6H and Supplementary Fig. 5), thus implying that JAKi are capable of variably skewing the inflammatory differentiation of macrophages independently of their fine specificity.

## Discussion

JAK inhibitors (JAKi) are targeted synthetic DMARD approved for the treatment of RA and other immune-mediated diseases, and target JAK-dependent signaling pathways of cytokines involved in inflammatory processes and immune regulation [21]. In the present manuscript we have evaluated the influence of clinically used doses of the JAKi Upadacitinib and Baricitinib on the GM-CSF-driven monocyte-to-macrophage differentiation. Our results indicate that JAKi not only weaken the expression of genes and proteins that characterize GM-CSF-dependent pro-inflammatory macrophages (activin A, *IL1B*) but also promote macrophage re-programming via enhanced expression of MAFB and MAFB-dependent genes (CD163, IL-10, LGMN) and the acquisition of an anti-inflammatory transcriptional and functional profile in vitro. The rise in MAFB and MAFB-dependent genes in Upa-treated macrophages correlates with increased of Ser-9 inactivating phosphorylation of GSK3β. However, the link between JAK2 inhibition and GSK3β inactivation remains unclear. We speculate that JAK2 inhibition affects Ser-9 GSK3β phosphorylation through PI3K-Akt or ERK, as these kinases are phosphorylated by JAK2 and target GSK3β at Ser-9. Alternatively, Upadacitinib may boost MAFB protein levels via ERK, which primes GSK3β-mediated phosphorylation and degradation of MAFB [52, 53]. In any event, since ERK inhibition alone does not raise MAFB levels (Fig. 5I), the higher MAFB levels in Upa-treated macrophages would be only partly mediated by the JAK2-ERK axis.

The pathological relevance of the re-programming effect of JAKi on human macrophages is supported by their effects on the expression of genes that define macrophage subsets specifically involved in tissue homeostasis and inflammation resolution. The analysis of synovial tissue macrophages from RA patients has previously identified inflammatory macrophage subsets (MerTK^pos^ and MerTK^neg^) which exhibit distinct gene profiles and whose presence is modulated during remission [3, 6]. Whereas tissue-resident MerTK^pos^ macrophages include TREM2^high^ macrophages in the protecting lining and FOLR2^high^LYVE1^pos^ macrophages in the sublining layer, the latter showing a unique remission transcriptomic signature, the tissue infiltrating MerTK^neg^ macrophage subset exhibits inflammatory functions [3]. Remarkably, we have found that Upadacitinib and Baricitinib favor the acquisition of genes that define the macrophage subsets TREM2^pos^ and FOLR2^high^LYVE1^pos^, primarily related to homeostasis and resolution of inflammation, thus supporting the therapeutic effect of JAKi on synovial macrophages [3]. Thus, as a whole, the JAK inhibitors Upadacitinib and Baricitinib limit the GM-CSF pro-inflammatory differentiation profile, block the inhibitory action of GM-CSF on anti-inflammatory pathways and drive a MAFB-dependent macrophage re-education.

In agreement with previous reports on cytokine-stimulated STAT phosphorylation [26], our analysis of JAKi-treated macrophages has revealed a dose-dependent effect of Upadacitinib on STAT5 phosphorylation, on modulation of STAT5-, GM-CSF- and M-CSF-dependent genes and proteins, on the macrophage re-direction towards an anti-inflammatory profile and on the expression of genes that characterize certain synovial macrophage subsets. On the other hand, it is worth noting that although all JAKi approved for RA treatment exhibit different specificity (Upadacitinib, a JAK1 inhibitor with effects on JAK2; Baricitinib, primarily a JAK1/2 inhibitor; Peficitinib, inhibitor of JAK3 over JAK1/2, and Filgotinib, primarily a JAK1 inhibitor), they all share a similar ability to re-direct macrophage differentiation and to dose-dependently induce MAFB and CD163 expression in GM-CSF-treated monocytes. The significance of this finding is further illustrated by lack of effect of Deucravacitinib, a TYK2 inhibitor, on the expression of MAFB and CD163 in pro-inflammatory monocytes.

Althought JAKi are generally well tolerated and exhibit a comparable safety profile to other bDMARDs [20, 24], a small increase in the risk of herpes zoster infection and other viruses have been observed in JAKi-treated patients [54, 55]. The viral reactivation is likely based on the ability of JAKi to block interferon I and II pathways [56]. Consequently, the effect of JAKi on the expression of MAFB is of particular interest because MAFB is known to modulate the efficiency of interferon production by setting a threshold for IRF3-dependent transcription [57]. Thus, our data suggest that the JAKi-dependent expression of MAFB might not be limited to macrophage re-programming towards an anti-inflammatory/homeostatic profile (CD163^+^FOLR2^+^IL10^+^) but contribute to vulnerability to viral infections through interferon regulation, an issue that deserves further investigation.

Our findings on the JAKi ability to trigger MAFB-dependent macrophage re-programming is reminiscent of the capacity of other drugs used for RA treatment to modulate MAFB expression. As an example, we have previously shown that high-dose methotrexate alters macrophage re-programming and downregulates the expression of MAFB [58]. Similarly, glucocorticoids, potent anti-inflammatory agents, skew monocyte differentiation in RA joints in a MAFB-dependent manner and via a transcriptional mechanism involving the binding of the glucocorticoid receptor (GR) to MAFB gene regulatory regions [59]. Specifically, GR binds both the promoter and enhancer regions within the *MAFB* gene, which is quickly upregulated and triggers the transcriptomic and epigenomic remodeling that gives rise to tolerogenic monocyte-derived dendritic cells. Given the significance of this mechanism, it might be of interest to determine whether GR is involved in the JAKi-induced MAFB enhanced expression. In this regard, we have observed that JAK2 inhibition by Upadacitinib results in upregulation of MAFB protein expression, an effect not seen after inhibition of either STAT5 or MEK (Fig. 5I). This result suggests that JAKi might alter macrophage differentiation by affecting additional intracellular signaling molecules. Indeed, we have found a correlation between the inhibitory Ser9 phosphorylation of GSK3β [60] and MAFB levels in JAKi-treated monocytes and macrophages (Figs. 5D, 6D). Since MAFB protein levels and activity is regulated by GSK3β [51, 53], and considering that GSK3β inhibition has an anti-inflammatory effect during chronic inflammation and ameliorates the clinical signs and tissue damage in the collagen-induced arthritis mouse model [61, 62], it is tempting to hypothesize that modulation of GSK3β activity might contribute to the JAK2-dependent human macrophage re-programming ability of JAKi. In any event, and regardless their potential action of GSK3β, our data reveal that JAKi re-direct macrophage differentiation towards the acquisition of a more anti-inflammatory/pro-resolution profile, an effect that correlates with the ability of JAKi to enhance MAFB expression.

### Supplementary Information

Below is the link to the electronic supplementary material.

Supplementary file1 (DOCX 15 KB)

Supplementary file2 (PDF 8433 KB)

Supplementary file3 (PDF 2495 KB)

Supplementary file4 (PDF 907 KB)

Supplementary file5 (PDF 1075 KB)

Supplementary file6 (PDF 870 KB)

Supplementary file7 (XLSX 138 KB)

Supplementary file8 (AVI 9443 KB)

## Data Availability

The dataset supporting the conclusions of this article is available in the Gene Expression Omnibus repository (http://www.ncbi.nlm.nih.gov/geo/) under accession number GSE232044 and GSE253495.
