# Supplementary material for: Macrophage re-programming by JAK inhibitors relies on MAFB
Source: Cell Mol Life Sci. 2024 Mar 25;81(1):152. doi: 10.1007/s00018-024-05196-1 (PMC10963568; doi:10.1007/s00018-024-05196-1)
Supplement: Supplementary file 1 — Supplementary file1 (DOCX 15 KB) [file 18_2024_5196_MOESM1_ESM.docx]

**SUPPLEMENTARY FILE**

**MATERIALS AND METHODS**

**Clinical and demographic data of patients included in the study**

**Patient 1**: A 50-year-old Caucasian woman with a 10-year history of seropositive rheumatoid arthritis (ACPA >3200 and rheumatoid factor positive at a titer of 1:80). She started conventional DMARDs including sulfasalazine hydroxychloroquine, methotrexate, and leflunomide and TNFi in June 2021 for persistent disease activity and radiographic progression. She experienced acceptable disease activity control with TNFi, but needed to suspend the biologic therapy in October 2021 for feet surgery. When TNFi was reinitiated, she experienced secondary failure and started upacitinib (rinvoq 15mg daily) in September 2022. General examination at that time revealed a pain on a visual analogue (VAS) of 7, patient global assessment VAS of 6, 3 tender joints, and 2 swollen joints. Laboratory investigations revealed a CRP of 18.4mg/L and ESR of 29 mm/h with a DAS28-CRP of 4.23 (moderate disease activity). She experienced a rapid control of inflammation being in remission by DAS-CRP in the last visit in May 2023.

**Patient 2**: A 59-year-old Caucasian woman with a 3-year history of seropositive rheumatoid arthritis (ACPA >3200 and rheumatoid factor positive at a titer of 1:756) and Cushing disease secondary to pituitary adenoma treated with neurosurgery and radiation therapy. She started methotrexate (MTX) in July 2020 and the dose was increased up to 15mg weekly with control of disease activity. In February of 2022 MTX was suspended for splenic and hepatic lesions in CT scan suspicious for lymphoproliferative disease. She started treatment with prednisone and hydroxychloroquine with poor control of disease activity. After ruling out cancer with an extensive study, she started upacitinib (rinvoq 15mg daily) in February 2023. General examination at that time revealed a pain on a visual analogue (VAS) of 5, a patient global assessment on a VAS of 7, 5 tender joints, and 4 swollen joints. Laboratory investigations revealed a CRP of 25.3mg/L and ESR of 22 mm/h with a DAS28-CRP of 4.93 (moderate disease activity). She experienced good and rapid response being in remission at the 3 months follow up.

**Patient 3**: A 28-year-old Hispanic woman with a 2-year history of seropositive rheumatoid arthritis (ACPA 359.9 and rheumatoid factor positive at a titer of 1:49). She started (MTX) in April 2021 with rapid scalation of dose. She experienced important side effects when using 20 mg/weekly, mainly nauseas, abdominal pain and hair loss, therefore MTX was reduced to 15mg weekly. In September 2022, she was admitted for a surgery procedure and MTX was suspended. After recovery, she did not reinitiated MTX and suffered a flare and prednisona was prescribed and MTX reinitiated. Adherence was poor because of side effects and the patient did not want to switch from oral to subcutaneous expressing needle phobia. In May 2023 she experienced a flare with morning stiffness of 1 hour, and 3 tender and 3 swollen joints (dominant hand) with a patient global assessment of 5, CRP and ESR were in normal range, and the patient suffered considerable limitation to perform her work duties. She then was started on upacitinib (rinvoq 15mg daily) with complete resolution of symptoms in the 3 months follow-up.

Nine sex-matched normal donors were selected after exclusion of autoimmune disease, neoplasia or active infection.
