# Supplementary material for: Macrophage re-programming by JAK inhibitors relies on MAFB
Source: Cell Mol Life Sci. 2024 Mar 25;81(1):152. doi: 10.1007/s00018-024-05196-1 (PMC10963568; doi:10.1007/s00018-024-05196-1)
Supplement: Supplementary file 2 — Supplementary file2 (PDF 8433 KB) [file 18_2024_5196_MOESM2_ESM.pdf]

Supplementary Figure 1

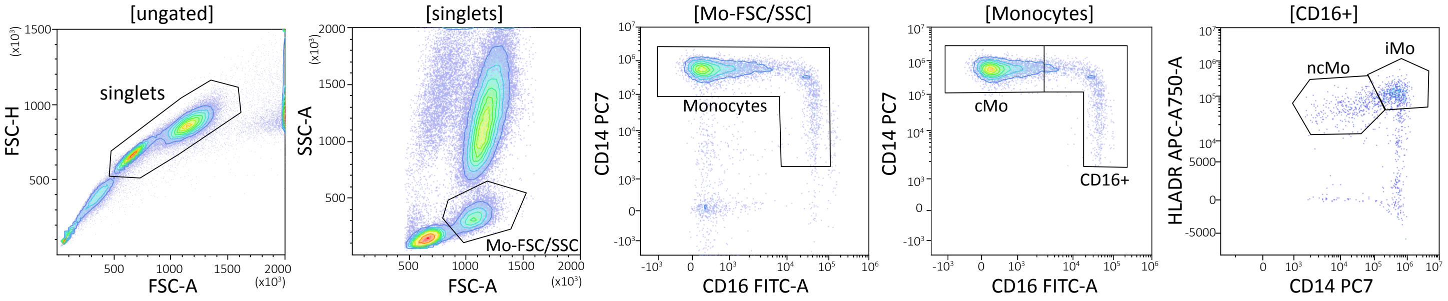

Supplementary Figure 1.- Gating strategy of the three monocyte subsets based on relative CD14, CD16 and HLADR expression. Monocytes were gated on the basis of singlets, forward scatter (FSC) and side scatter (SSC) and CD14/CD16 expression. Monocyte susbsets were identified as follows: Classical monocytes (cMo) were gated as CD14++ CD16- in the CD14/CD16 plot; the resulting CD16+ monocytes were analyzed by HLADR/CD14 expression, resulting in intermediate monocytes (iMo, CD14++HLA-DR++) and non-classical monocytes (ncMo, CD14+HLADR+).
