# Supplementary material for: Macrophage re-programming by JAK inhibitors relies on MAFB
Source: Cell Mol Life Sci. 2024 Mar 25;81(1):152. doi: 10.1007/s00018-024-05196-1 (PMC10963568; doi:10.1007/s00018-024-05196-1)
Supplement: Supplementary file 3 — Supplementary file3 (PDF 2495 KB) [file 18_2024_5196_MOESM3_ESM.pdf]

Supplementary Figure 2

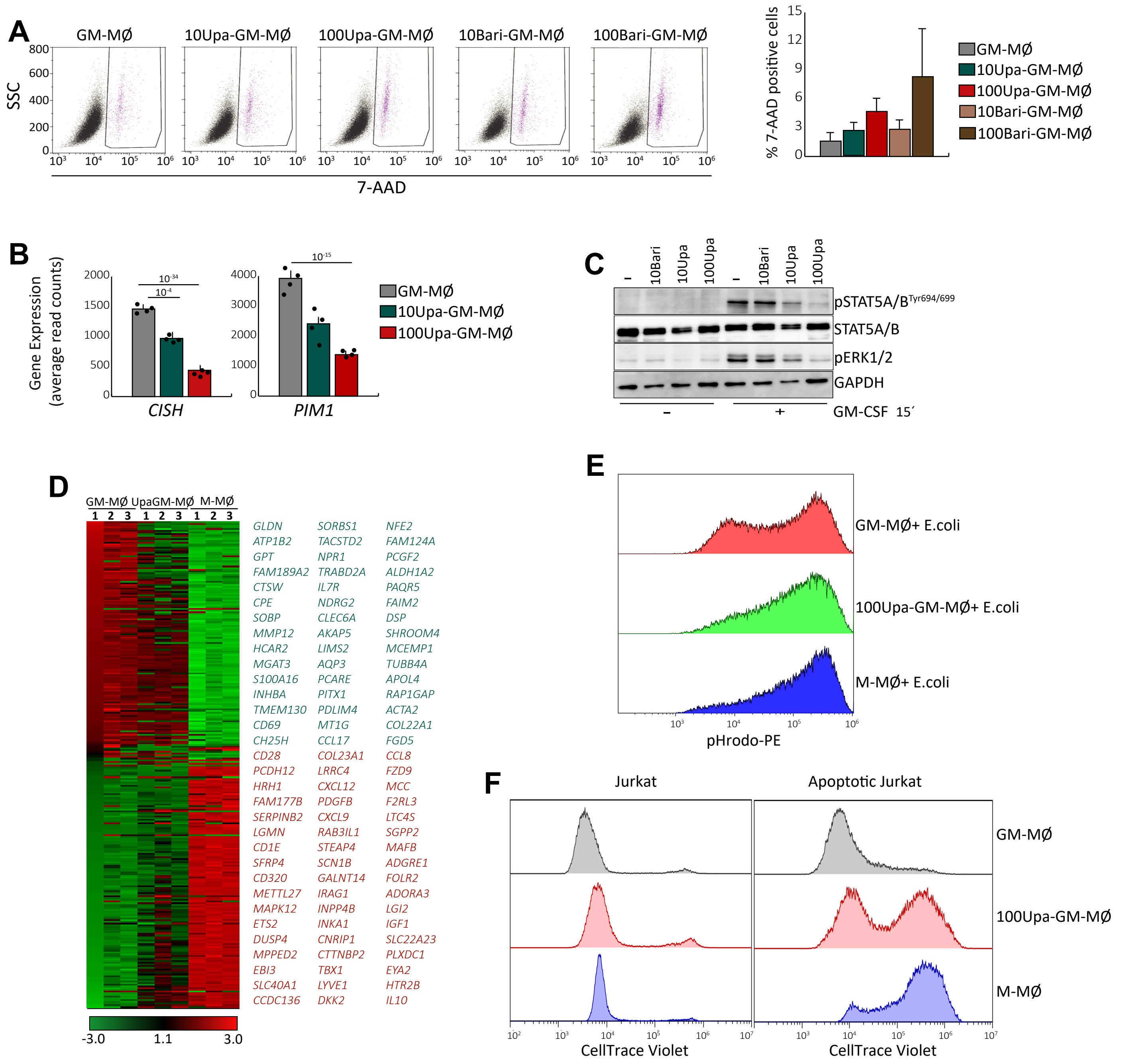

Supplementary Figure 2.- Effect of JAK inhibitors on macrophage viability, STAT5-dependent genes, STAT5 phosphorylation levels, phagocytosis and efferocytosis (A) Cellular viability, as determined by flow-cytometry using 7-aminoactinomycin D (7-AAD) in GM-MØ, 10Upa-GM-MØ, 100Upa-GM-MØ, 10Bari-GM-MØ and 100Bari-GM-MØ. A representative experiment is shown. Right, quantification of 7-AAD-positive cells in the indicated JAKi-exposed GM-MØ, as determined by flow-cytometry. Mean ± SEM of three independent experiments are shown. **(B)** Relative expression of the indicated STAT5-dependent genes as determined by RNA-sequencing on GM-MØ, 10Upa-GM-MØ and 100Upa-GM-MØ. Mean ± SEM of 4 independent donors are shown, with the indication of the *P*adj.**(C)** Immunoblot analysis of pSTAT5, STAT5, pERK and ERK by monocytes treated for 1h to DMSO (-), 10 nM Baricitinib (10Bari), 10 nM Upadacitinib (10Upa) or 100 nM Upadacitinib (100Upa) and exposed to GM-CSF for 15 min. GAPDH protein levels were determined as protein loading controls. A representative experiment of two independent donors is shown. **(D)** Heatmap of the expression of M-MØ-specific and GM-MØ-specific genes in GM-MØ, 100Upa-GM-MØ (from GSE232044) and M-MØ (data extracted from GSE186151). For each gene, mRNA expression level is represented after normalizing gene expression and k-means clustering using Genesis (http://genome.tugraz.at/genesisclient/). **(E)** Phagocytic activity of GM-MØ, 100Upa-GM-MØ and M-MØ as determined by flow cytometry using pHrodo Red E. coli BioParticles Conjugates. A representative histogram for pHrodo emission of GM-MØ, 100Upa-GM-MØ and M-MØ is shown. **(F)** Efferocytosis of GM-MØ, 100Upa-GM-MØ and M-MØ as determined by flow cytometry using CellTrace Violet-labeled Jurkat cells (left) or staurosporine-induced CellTrace Violet-labeled apoptotic Jurkat cells (right). A representative histogram for CellTrace Violet emission of GM-MØ, 100Upa-GM-MØ and M-MØ is shown.
