# Supplementary material for: Macrophage re-programming by JAK inhibitors relies on MAFB
Source: Cell Mol Life Sci. 2024 Mar 25;81(1):152. doi: 10.1007/s00018-024-05196-1 (PMC10963568; doi:10.1007/s00018-024-05196-1)
Supplement: Supplementary file 4 — Supplementary file4 (PDF 907 KB) [file 18_2024_5196_MOESM4_ESM.pdf]

Supplementary Figure 3

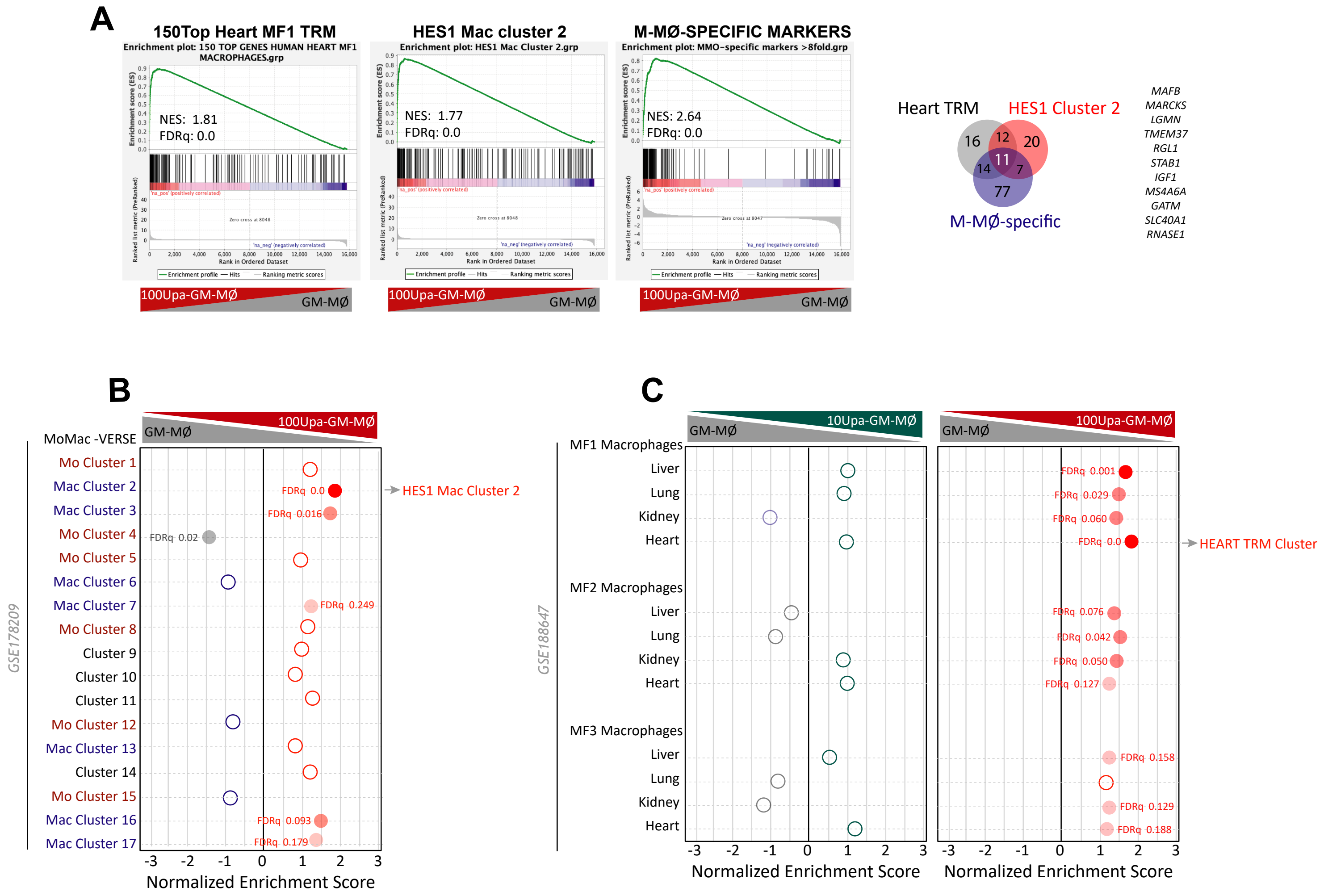

Supplementary Figure 3.- Upadacitinib modulates the expression of genes that define tissue-resident macrophage (A) GSEA on the ranked comparison of the GM-MØ versus 100Upa-GM-MØ transcriptomes, using the genes preferentially expressed by Heart macrophage cluster 1 (MF1) Tissue Resident Macrophages (TRM) (GSE188647), the genes preferentially expressed by HES1 Mac specific cluster 2 of tissue resident macrophages from the MoMac-VERSE (GSE178209), and the genes significantly modulated by M-CSF (M-MØ-specific markers) (GSE188278) as data set. Normalized Enrichment Score (NES) and False Discovery rate q value (FDRq) are indicated. Right, comparison of genes differentially expressed in the indicated macrophage types. **(B)** GSEA on the ranked comparison of the GM-MØ versus 100Upa-GM-MØ transcriptomes, using the monocyte and macrophage cluster defined in the MoMac-VERSE as data set. Normalized Enrichment Score and False Discovery rate q value (FDRq) are indicated. The intensity of color increases with the enrichment of the gene signature. **(C)** GSEA on the ranked comparison of the GM-MØ versus 10Upa-GM-MØ and GM-MØ versus 100Upa-GM-MØ transcriptomes, using the MF1, MF2 and MF3 clusters of tissue resident macrophages from liver, lung, kidney and heart (GSE188647) as data set. Normalized Enrichment Score and False Discovery rate q value (FDRq) are indicated. The intensity of color increases with the enrichment of the gene signature.
