# Supplementary material for: Macrophage re-programming by JAK inhibitors relies on MAFB
Source: Cell Mol Life Sci. 2024 Mar 25;81(1):152. doi: 10.1007/s00018-024-05196-1 (PMC10963568; doi:10.1007/s00018-024-05196-1)
Supplement: Supplementary file 5 — Supplementary file5 (PDF 1075 KB) [file 18_2024_5196_MOESM5_ESM.pdf]

Supplementary Figure 4

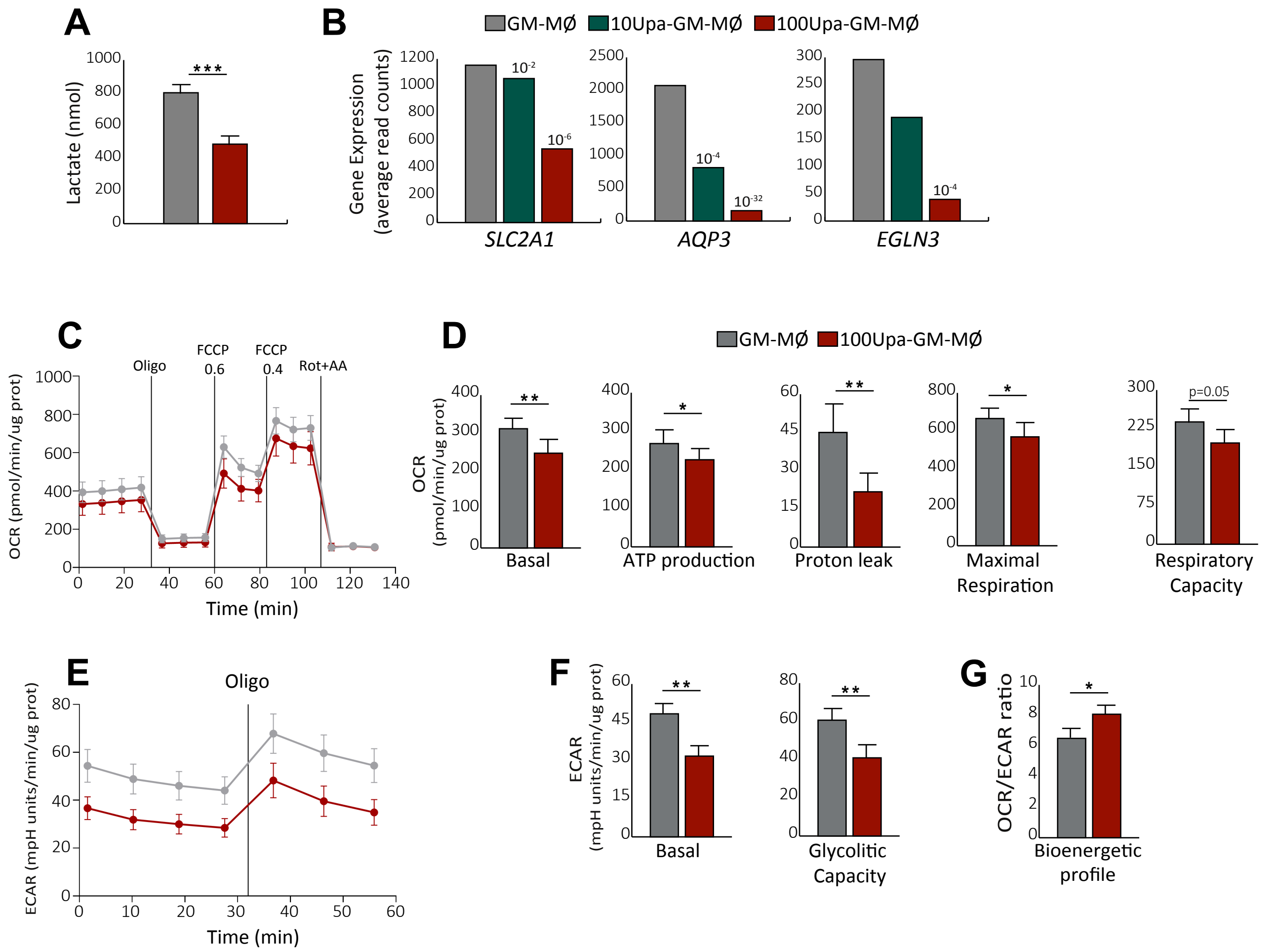

Supplementary Figure 4.- Lactate, HIF1A-dependent gene expression and characterization of the energy metabolism in Upadacitinib-treated macrophages (A) Production of lactate by GM-MØ and 100Upa-GM-MØ. Mean ± SEM of 16 independent donors (****p*<0.001, paired t-test). **(B)** Relative level of expression of the indicated genes as determined by RNA-sequencing on GM-MØ, 10Upa-GM-MØ and 100Upa-GM-MØ. **(C)** OCR profile of human GM-MØ and 100Upa-GM-MØ monitored using the Seahorse Biosciences extracellular flux analyzer. Cells were treated sequentially, as indicated, with 1 μM oligomycin (Oligo), 0.6 plus 0.4 μM FCCP, and 1 μM rotenone plus 1 μM antimycin A (Rot/AA). **(D)** Metabolic parameters obtained from the OCR profiling after subtraction of the rotenone/antimycin-insensitive respiration. Basal OCR is the oxygen consumption rate in the absence of effectors, ATP turnover is considered as the oligomycin-sensitive respiration, and maximal respiration is the OCR value in the presence of the uncoupler FCCP. Results are normalized according to protein concentrations and presented as mean ± SEM of 6 independent samples. **(E)** ECAR, a proxy for the rate of lactate production, measured in human GM-MØ and 100Upa-GM-MØ under basal conditions and after the stimulation with 1 μM oligomycin. **(F)** Metabolic parameters obtained from ECAR profiling. Basal indicates the rate of glycolysis in the absence of effectors, and glycolytic capacity is the ECAR value after the inhibition with oligomycin of the mitochondrial ATP synthesis. Results are normalized according to protein concentrations and presented as mean ± SEM of 6 independent samples. **(G)** The bioenergertics profile of the cells is defined as the ratio between the basal OCR and basal ECAR. Results are presented as mean ± SEM of 6 independent samples (**p*<0.05***p*<0.01, paired t-test).
