# Supplementary material for: Macrophage re-programming by JAK inhibitors relies on MAFB
Source: Cell Mol Life Sci. 2024 Mar 25;81(1):152. doi: 10.1007/s00018-024-05196-1 (PMC10963568; doi:10.1007/s00018-024-05196-1)
Supplement: Supplementary file 6 — Supplementary file6 (PDF 870 KB) [file 18_2024_5196_MOESM6_ESM.pdf]

Supplementary Figure 5

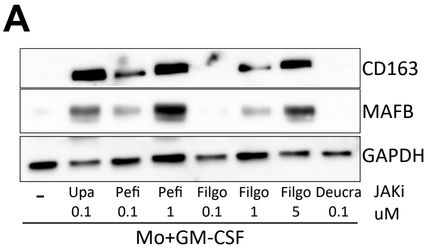

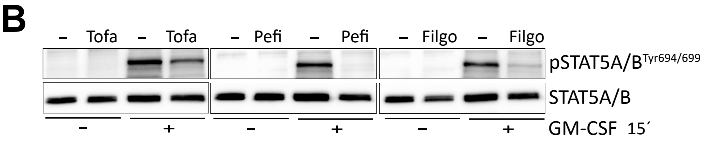

Supplementary Figure 5.- Effect of JAK inhibitors Peficitinib, Filgotinib and Deucravacitinib on MAFB, CD163 expression and STAT5 phsophorylation levels (A) Immunoblot analysis of MAFB and CD163 in GM-MØ (day 2) generated from monocytes exposed to DMSO (-) or the indicated concentrations of Upadacitinib (Upa), Peficitinib (Pefi), Filgotinib (Filgo) or Deucravacitinib (Deucra). A representative experiment of two independent donors is shown. **(B)** Immunoblot analysis of pSTAT5 and STAT5 by monocytes treated for 1h to DMSO (-), 100 nM Tofacitinib (Tofa), 1 uM Peficitinib (Pefi) or 5 uM Filgotinib (Filgo) and exposed to GM-CSF for 15 min. The concentrations were selected based on *in vivo* dosage. A representative experiment of two independent donors is shown.
